# Exploring Overall and Component Complexities via Relative Complexity Change and Interacting Complexity Amplitudes in the Kolmogorov Plane: A Case Study of U.S. Rivers

**DOI:** 10.3390/e27101006

**Published:** 2025-09-26

**Authors:** Dragutin T. Mihailović, Slavica Malinović-Milićević

**Affiliations:** 1 Faculty of Natural Sciences, Department of Physics, University of Novi Sad, 21000 Novi Sad, Serbia; 2 Geographical Institute “Jovan Cvijić” SASA, 9 Đure Jakšića St., 11000 Belgrade, Serbia; s.malinovic-milicevic@gi.sanu.ac.rs; 3 Institute of Environmental Engineering, Peoples’ Friendship University of Russia (RUDN University), 6 Miklukho-Maklaya St., 117198 Moscow, Russia

**Keywords:** streamflow complexity, Kolmogorov complexity (KC), Kolmogorov complexity spectrum (KC spectrum), Kolmogorov complexity plane (KC plane), relative change of complexity (RCC), histogram of RCC, U.S. rivers

## Abstract

One of the most challenging tasks in studying streamflow is quantifying how the complexities of environmental and dynamic parameters contribute to the overall system complexity. To address this, we employed Kolmogorov complexity (KC) metrics, specifically the Kolmogorov complexity spectrum (KC spectrum) and the Kolmogorov complexity plane (KC plane). These measures were applied to monthly streamflow time series averaged across 1879 gauge stations on U.S. rivers over the period 1950–2015. The variables analyzed included streamflow as a complex physical system, along with its key components: temperature, precipitation, and the Lyapunov exponent (LEX), which represents river dynamics. Using these metrics, we calculated normalized KC spectra for each position within the KC plane, visualizing interactive master amplitudes alongside individual amplitudes on overlapping two-dimensional planes. We further computed the relative change in complexities (RCC) of the normalized master and individual components within the KC plane, ranging from 0 to 1 in defined intervals. Based on these results, we analyzed and discussed the complexity patterns of U.S. rivers corresponding to each interval of normalized amplitudes.

## 1. Introduction

Quantifying the overall complexity of systems and understanding the contributions of their components involves diverse methodologies spanning engineering, software, and theoretical frameworks. Concerning this, we list several approaches that address these aspects.

*Structural and integrative complexity*. Structural complexity refers to the complexity arising from the number and arrangement of a system’s elements and their interrelationships. It focuses on how many different ways system components can be combined and connected, which directly impacts the system’s potential adaptability and behavior. Riesener et al. [1] proposed a structural complexity framework for cyber-physical systems, emphasizing three factors: component count, interaction density, and topology. It quantifies overall complexity through these structural properties. Integrative complexity is defined as the difference between the total system complexity and the sum of the complexities of its individual modules, based on a given system decomposition. It quantifies the complexity involved in integrating or assembling the system from its constituent modules [2].

*Component-based metrics*. This method refers to quantitative measures used to evaluate various attributes of software components and component-based software systems. These metrics help assess aspects such as complexity, quality, reusability, coupling, cohesion, integration, and overall system maintainability. Kumari and Upadhyaya [3] developed an average interface interaction complexity metric for software components. It calculates complexity via input/output interactions, linking component interfaces to system-wide complexity. Gomes et al. [4] introduced a system complexity metric that maps the internal connections of a system through its dynamic states and evaluates how each parameter contributes to output variability using sensitivity indices. This methodology includes three main steps: (a) Calculation of parameter sensitivity indices to assess the influence of each input on system outputs. (b) Mapping the dynamic connections between system components based on system states. (c) Computing the *overall system complexity* by integrating connection mapping with sensitivity information.

*Information-theoretic approaches*. These approaches provide a general framework for estimating both overall and component complexities in complex systems by quantifying how information is stored, transferred, and modified within the system. These methods translate a system’s mechanistic description into an informational description, enabling the analysis of multi-scale structure and dynamics across components and their interactions [5,6,7].

The relationship between streamflow complexity and the complexity of environmental factors is a critical issue in hydrological research, especially in the context of changing climatic conditions. Numerous studies have examined this relationship by analyzing the complexity of both environmental parameters and streamflow dynamics, often using entropy-based measures or traditional statistical methods [8,9,10], among others. These investigations have revealed that streamflow complexity is influenced by a combination of natural processes—such as precipitation, temperature variability, and land surface changes—and human activities, which can significantly alter the predictability and randomness of hydrological systems. While entropy and complexity metrics have been effective in characterizing these dynamics, challenges remain due to the nonlinearity and multi-scale nature of the interactions, highlighting the need for integrated approaches to better understand and model streamflow behavior under environmental variability. In contrast to conventional methods, this challenge can be effectively addressed using an alternative framework that leverages two innovative complexity measures: the Kolmogorov complexity spectrum (KC spectrum) [11] and the Kolmogorov complexity plane of interacting amplitudes (KC plane) [12]. The KC plane enables the identification of specific intervals of interacting complexity amplitudes, allowing for the quantification of how complexities in individual meteorological elements and dynamic processes contribute to the overall complexity of streamflow. Exploring complexity via relative complexity change and interacting complexity amplitudes in the KC plane offers a more comprehensive, dynamic, and interaction-sensitive analysis compared to traditional methods that may be limited by predefined features, static views, or statistical assumptions. The method proposed in this study falls within the domain of information-theoretic approaches, focusing on the relationship between the overall complexity of a complex system and the complexities of its individual components.

This paper examines the relationship between the overall complexity of streamflow in U.S. rivers from 1950 to 2015 and the combined complexities of environmental factors (temperature and precipitation) alongside dynamical components (Lyapunov exponent) that characterize streamflow as a complex physical system. The paper is organized as follows: Section 2 describes the data sources; Section 3 provides a detailed description of the methodology; Section 4 presents and discusses the results; and Section 5 summarizes the key conclusions drawn from the study.

## 2. Description of Data Used

**Figure 1:**
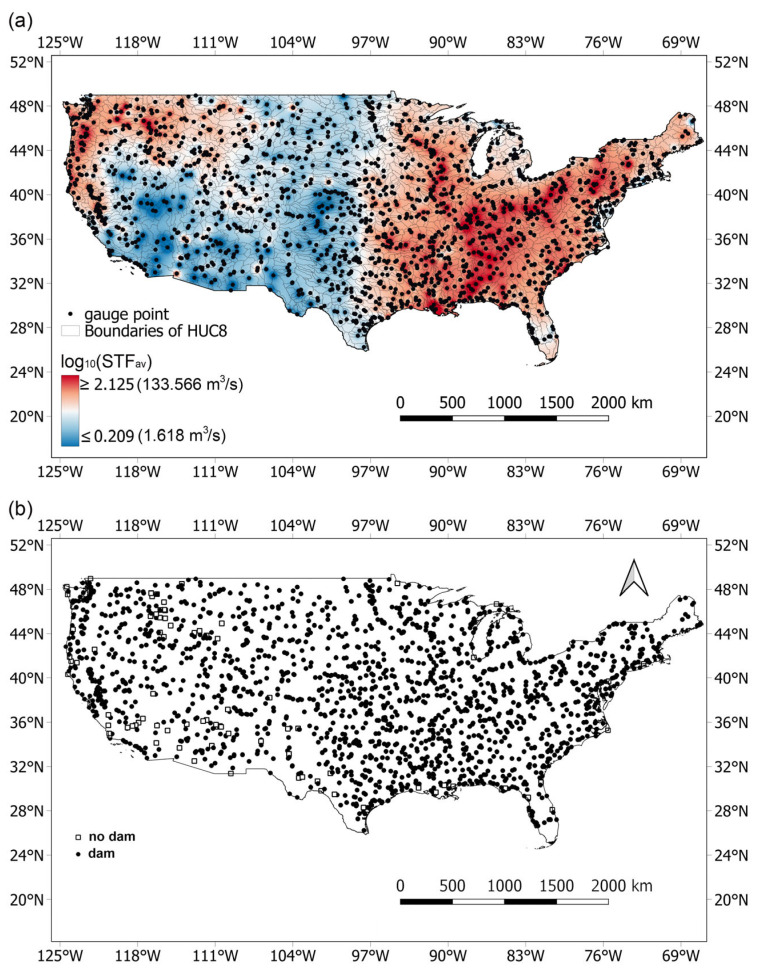
Spatial distributions of (**a**) maximum streamflow (m^3^/s), and (**b**) dam locations across U.S. rivers for the period 1950–2015. Reprinted from [16] with permission. Copyright 2023 Elsevier.

Data on monthly naturalized streamflow from 1950 to 2015 were obtained from the U.S. Geological Survey (USGS) Science Base Catalog. This dataset consists of simulated streamflow values for 2,622,273 stream reaches defined by the National Hydrography Dataset (NHD) Version 2.0, covering the continental United States. The simulations were generated using a random forest ensemble approach [13]. However, utilizing the entire dataset of over 2.5 million stream reaches would introduce redundant information and increase the risk of including less reliable estimates. This is because the random forest models were calibrated using observed streamflow data from approximately 2000 reference gauge sites and then applied to ungauged stream segments [13]. To ensure data quality and relevance, we limited our analysis to naturalized streamflow data from reaches directly connected to gauge stations located at the outlets of Hydrologic Unit Code 8 (HUC8) watersheds, resulting in a subset of 1879 sites (Figure 1a). Annual mean precipitation and temperature for each of these 1879 sites were derived from the NOAA nClimGrid monthly dataset. Covering the period from 1895 to the present, the nClimGrid provides data for the contiguous United States (CONUS) and Alaska at a 5 km spatial resolution [14]. For the overlapping period of 1950–2015, precipitation and temperature values were spatially interpolated to the naturalized streamflow sites using the inverse distance weighting (IDW) method [15]. In addition, the locations of 92,075 dams across CONUS were obtained from the National Inventory of Dams (NID), and dam locations across U.S. rivers for the period 1950–2015 (Figure 1b).

Locations across U.S. rivers were analyzed for the period 1950–2015. The dataset consists of monthly averaged values for streamflow (STF), temperature (TAV), precipitation (PRE), and the Lyapunov exponent (LEX), aggregated from multiple gauge stations. In total, each time series contains 792 monthly samples, representing the average conditions for each parameter from January 1950 through December 2015. This aggregation enabled a consistent and comprehensive analysis of hydrological and climatic variables over the 66-year period. Time series formed in this way provide a solid foundation for exploring the overall complexity of streamflow as well as the complexities of its individual components—such as temperature and precipitation, which represent climatic variables, and the Lyapunov exponent, which reflects the chaotic dynamics of U.S. rivers—using the method proposed in this study.

Let us emphasize that this study primarily focuses on a method capable of determining the dependency and hierarchical relationship between overall complexity and its components. In this case, a hydrological series was constructed from a large number of individual hydrological time series. Naturally, this method is also applicable to other time series obtained from measurements in fields such as nuclear physics, neuroscience, psychiatry, and others, where establishing the dependency of overall complexity on its components is particularly challenging. Of course, the nature and formation of these dependencies vary significantly across different domains.

Descriptive statistics for the time series representing the mean monthly values of streamflow (STF), temperature (TAV), and precipitation (PRE) are summarized in Table 1 and briefly commented on below.

The STF time series shows a moderate right skew with a mean of 147.79 and a median of 134.77. It has a wide range from 22.01 to 456.66 and substantial variability, indicated by a standard deviation of 84.90. The interquartile range is moderately spread (Q1 = 70.48, Q3 = 211.23). Skewness (0.58) confirms a modest right tail, and kurtosis (2.58) suggests a slightly sharper peak than a normal distribution. On the whole, the series is moderately dispersed with a tendency toward higher values.

The TAV time series is nearly symmetric, with a mean of 12.33 close to the median of 12.69. It spans a moderate range from −4.53 to 25.71 and shows moderate variability, with a standard deviation of 8.41. The interquartile range, from 4.65 to 20.42, indicates a reasonably broad central spread. The skewness of −0.07 confirms the distribution is almost symmetric, while a kurtosis of 1.61 suggests the data is somewhat flatter than a normal distribution. Taken as a whole, the series is moderately dispersed with a balanced distribution shape.

The PRE time series is highly symmetric, with nearly identical mean and median values. It has a moderate range from 15.25 to 117.71 and shows moderate variability, with a standard deviation of 14.57. The interquartile range (58.19 to 76.33) indicates a fairly concentrated middle 50%. Skewness near zero (0.01) confirms symmetry, while a kurtosis of 3.29 suggests a slightly more peaked distribution. Overall, the series is moderately dispersed with a balanced and mildly leptokurtic shape.

The LEX time series is right-skewed, with a mean of 0.13 exceeding the median of 0.12 and a high skewness of 2.46. Values range narrowly from 0.10 to 0.25, showing low variability with a standard deviation of 0.03. The interquartile range is tight (Q1 = 0.12, Q3 = 0.13), indicating most values cluster near the lower quartiles. A high kurtosis of 8.13 points to heavy tails and potential outliers. Overall, the series is moderately dispersed but strongly skewed with possible extreme values.

There is indeed a known lack of standardization in defining and computing the Lyapunov exponent from empirical datasets due to differing methodological approaches. The main debate centers on whether to treat data longitudinally by tracking individual trajectories over time or cross-sectionally by analyzing ensembles of states at given times. In this study, the data sources were handled longitudinally. In the context of calculating the Lyapunov exponent, longitudinal tracking refers to tracking the divergence over time of trajectories in the reconstructed phase space starting from a single reference trajectory (such as an original time series) and its nearest neighboring trajectory. This involves repeatedly observing how the distance between initially close points evolves as they “flow” through the phase space over time, capturing the exponential separation that characterizes chaos and instability in the system. This approach is fundamental in algorithms like Rosenstein’s and Wolf’s methods for calculating the maximum Lyapunov exponent. We calculated the largest Lyapunov exponent (LEX) for the monthly streamflow time series by applying the Rosenstein algorithm [17]. This algorithm is a practical, fast, and robust method focused on estimating the largest Lyapunov exponent. It is well-suited for relatively short and noisy datasets, thanks to its simplicity and resilience to variations in parameter choices. The algorithm was implemented in MATLAB R2020a following the approach described by [18].

Given a time series $\begin{array}{cc} \left\{x_{i}\right\}, & i=1,\;2,\;3,\;4,\;\ldots ,N \end{array}$, normalization was performed using the transformation:

$$x_{i}=\frac{X_{i}-X_{min}}{X_{max}-X_{min}},$$

where $\left\{X_{i}\right\}$ represents the original observed time series, $X_{max}=max\left\{X_{i}\right\}$ is the maximum value in the series, and ${\;X}_{min}=min\left\{X_{i}\right\}$ is the minimum value. This normalization ensures all values fall within the range of 0 to 1.

**Figure 2:**
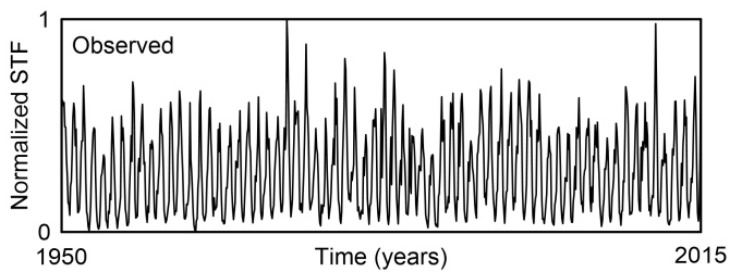
Time series of monthly streamflow values averaged over 1879 gauge stations on U.S. rivers for the period 1950–2015. All values are normalized.

**Figure 3:**
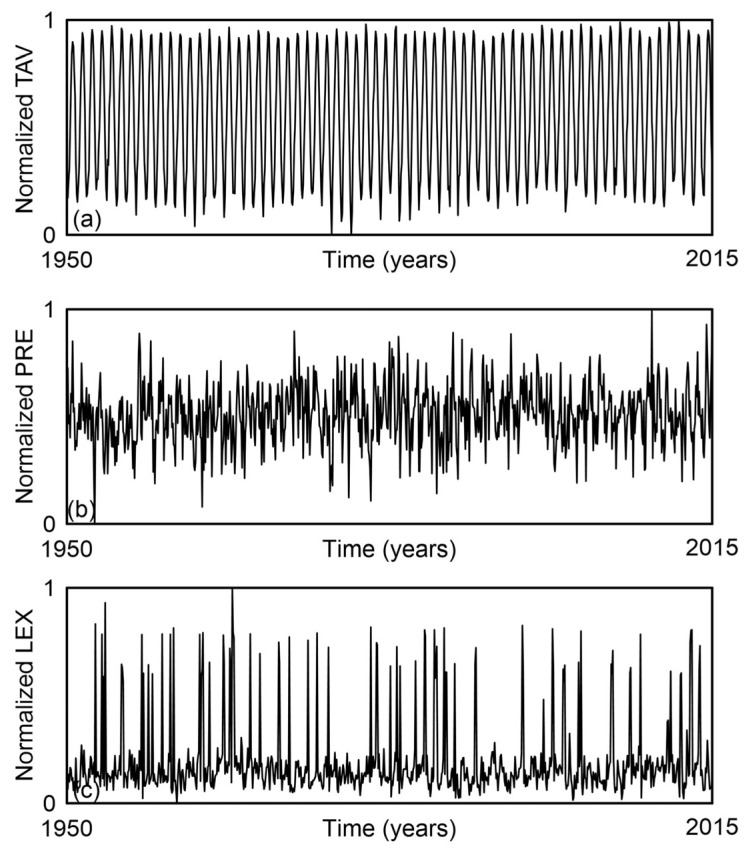
Time series of the mean monthly values (**a**) temperature (TAV), (**b**) precipitation (PRE), and (**c**) the Lyapunov exponent (LEX), averaged over 1879 gauge stations on U.S. rivers for the period 1950–2015. All values are normalized.

To compare the overall complexity of streamflow with the complexities of its individual components, we used normalized time series, shown in Figure 2 and Figure 3. Each time series consisted of N = 792 samples.

## 3. Description of Method

We describe a method for studying overall and component complexities using relative complexity change and interactive complexity amplitudes in the Kolmogorov complexity plane (KC plane). The approach involves the following steps: (1) calculating the Kolmogorov complexity spectrum (KC spectrum) for the quantity of interest; (2) constructing the KC plane to analyze interactions among components; and (3) computing the relative change of complexity (RCC) in the quantity of interest. In this study, the quantities of interest are streamflow (STF), temperature (TAV), precipitation (PRE), and the Lyapunov exponent (LEX).

### 3.1. Kolmogorov Complexity (KC)

It quantifies the randomness of an object by the length of the shortest program needed to reproduce it (Appendix A). It provides tools to analyze chaotic systems, optimize models, and enhance forecasting accuracy. By evaluating the complexity of datasets, researchers can reveal underlying patterns and dependencies that are often hidden in raw data. This insight enables the development of more effective and accurate models for weather and climate prediction. Specifically, a low Kolmogorov complexity suggests the presence of regular, predictable structures, while a high complexity indicates chaotic dynamics and reduced predictability. Since KC is non-computable for arbitrary objects, it is commonly approximated using compression methods like the Lempel–Ziv algorithm (LZA) [19]. A key limitation of LZA is its dependence on binary time series, requiring binarization that can cause information loss by oversimplifying the original data. Despite this, LZA remains popular due to its adaptability, simplicity, and asymptotic optimality without needing prior knowledge of source statistics.

### 3.2. Kolmogorov Complexity Spectrum (KC Spectrum)

**Figure 4:**
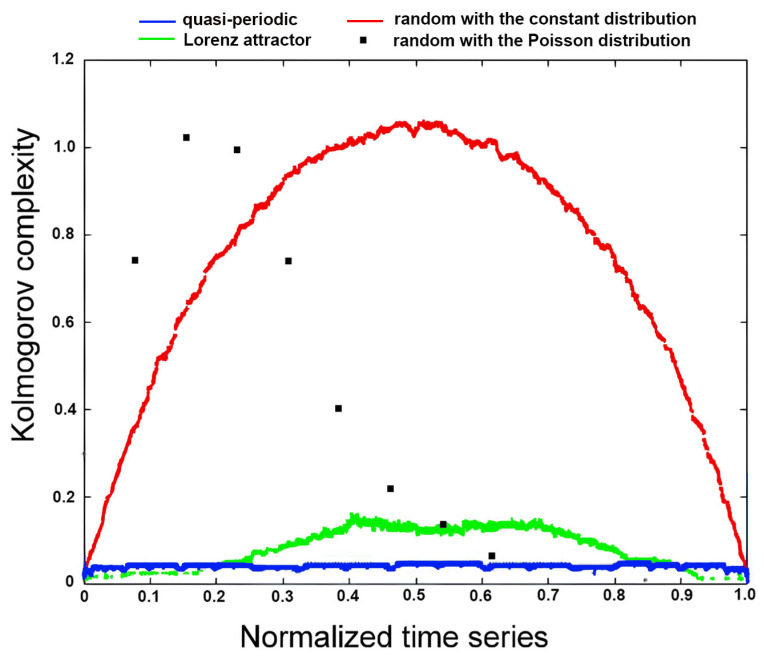
KC spectrum for different time series: quasi-periodic, Lorenz attractor, random with a uniform distribution, and random with a Poisson distribution (Figure courtesy of Marcelo Kovalsky).

This measure, introduced by Mihailović et al. [11], offers a novel framework for analyzing complex, stochastic systems. Figure 4 shows KC spectra for various time series—quasi-periodic, Lorenz attractor, and random series with uniform and Poisson distributions—demonstrating the method’s ability to distinguish chaotic and quasi-periodic dynamics from purely random processes. The KC spectrum captures the sensitivity of a time series’ structural complexity to local thresholds by treating each data point as a binarization threshold, enabling a multi-scale analysis of informational properties. The calculation procedure using the Lempel–Ziv algorithm (LZA) is detailed in Appendix A and further explained in [20].

### 3.3. Kolmogorov Complexity Plane (KC Plane) and Interacting Complexity Amplitudes

One of the most challenging tasks in studying complex physical systems is determining how the complexities of individual components contribute to the overall complexity of the entire system. The Kolmogorov complexity plane of interacting amplitudes (KC plane) is a conceptual framework used to analyze these contributions. This approach, introduced by Mihailović and Singh [12], employs the KC plane as a two-dimensional space bounded by (1,1) to study physical systems and their components through their respective complexities.

Before proceeding to the methodology for assessing how the complexities of individual components contribute to the overall complexity of the entire physical system, we will first clarify the terminology that will be used throughout the analysis. The specific term “master complexity” in complex systems of physics is not widely standardized or explicitly defined in common scientific literature, particularly within hydrological and engineering references. However, within the broader context of complex systems—including river flow and other physical systems—the term ’master complexity’ can be understood as an aggregated or holistic measure of the system’s complexity, synthesizing the complexities of individual components inherent in the system’s overall behavior.

In practical applications, master complexity is often quantified using information-theoretic measures such as KC, KC spectra, or related entropy-complexity metrics, which integrate multiple flow characteristics into a single comprehensive complexity measure. For example, a river flow system, as a complex system comprises both physical and ecological elements that interact dynamically. Streamflow serves as the master component, while individual components include environmental factors such as temperature, precipitation, and evapotranspiration.

**Figure 5:**
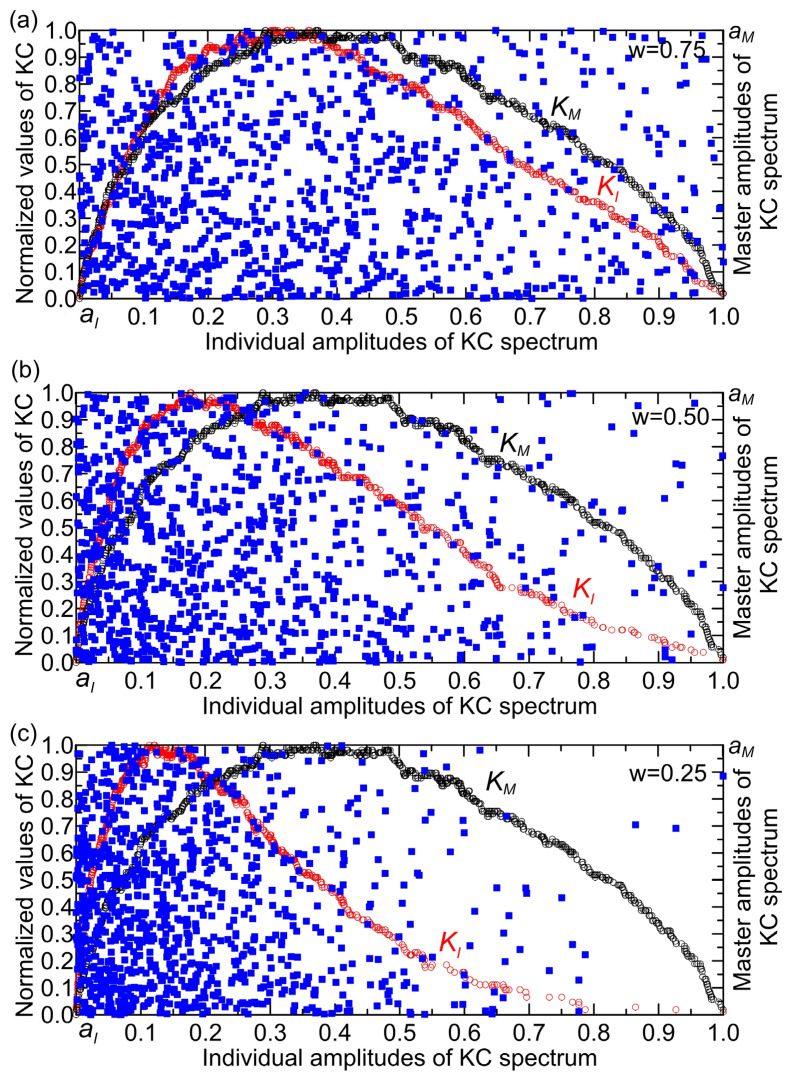
Mapping the ($K_{I},K_{M}$) coordinates in overlapping two-dimensional systems ($a_{I},KC$) and ($a_{I},a_{M}$)$\;for\;(a)\;w=0.75,\;(b)\;w=0.5,\;and\;(c)\;w=0.25.\;K_{I}$ and $K_{M}$ represent KC spectra for the individual system component and the master system component, respectively.

To illustrate the KC plane concept, we generate a time series $\left\{x_{i}\right\},\;i=1,\;2,\;3,\;\ldots ,\;N$ defined by $\left\{x_{i}\right\}=e^{-w\sigma_{i}}$, where w is an amplitude factor and $\sigma_{i}$ is uniformly distributed over [0, 1]. We create one master series with w=1.0 (component A) and three others with w= 0.75, 0.50, and 0.25 representing master and individual components (B, C, and D) of the KC spectra $K_{M}$ (black circles) and $K_{I}$ (red circles), respectively (Figure 5). In this example, component A can represent streamflow, while B, C, and D correspond to temperature, precipitation, and Lyapunov exponent, respectively.

Let us consider Figure 5, which shows two overlapping coordinate systems. In each system, the *x*-axis represents the amplitude of the KC spectra: $K_{I}$ for the individual system component and $K_{M}$ for the master system component. The *y*-axis represents the normalized KC values (left axis), while the right axis shows the master amplitude $a_{M}$ of the KC spectrum. Symbolically, we denote these two-dimensional systems as ($a_{I\;},KC$) and ($a_{I\;},a_{M}$). The set of points in this system, depicted as blue squares in Figure 5, is formed by elements taken from the time series of KC spectra $K_{I\;}$ and $K_{M}$, resulting in a set of  N points in the planes ($a_{I\;},KC$) and ($a_{I\;},a_{M\;}$). This representation effectively maps the spectral complexity amplitudes against their normalized values, facilitating analysis of their relationships within the time series data (Figure 5). For clarity, we note the following. As a result of the model presented, which involves normalization of the data and the function used, it appears that the values of the vertical axis ($a_{M}$) and the horizontal axis ($a_{I}$) are the same. Both axes, horizontal and vertical, carry the same normalized quantity, but the $a_{I}$-axis refers to the amplitudes of individual components of the system, while the $a_{M\;}$-axis refers to the amplitudes of the system’s master complexity.

**Figure 6:**
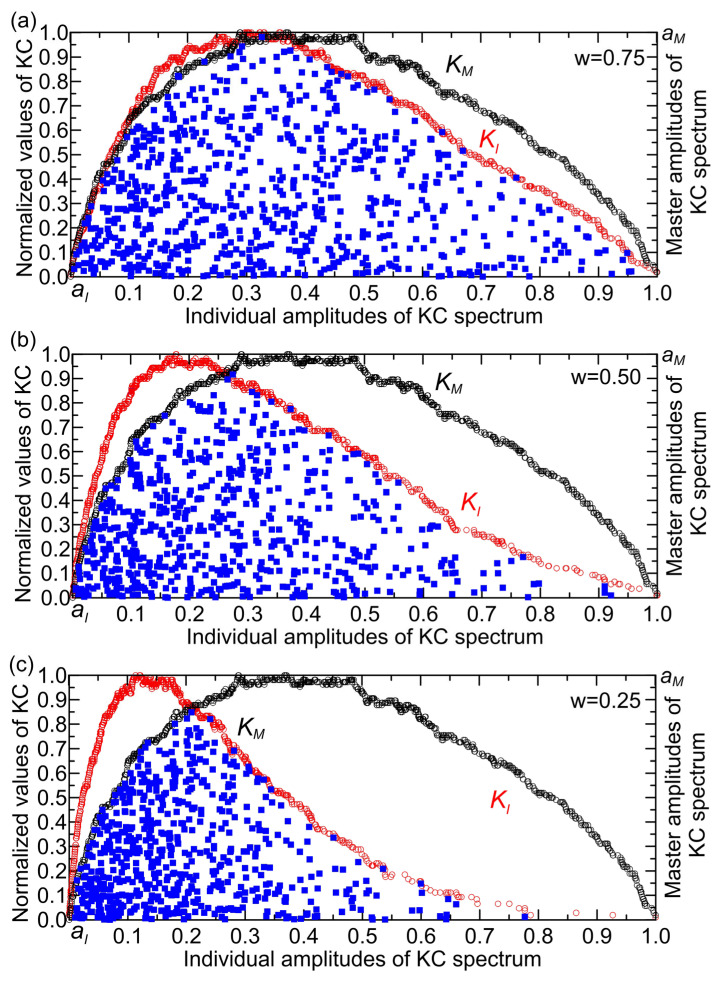
Defining the KC plane through interacting complexity amplitudes for (**a**) w = 0.75, (**b**) w = 0.50, and (**c**) w = 0.25.

However, interaction between the master and individual complexity amplitudes occurs only at points on the surface bounded by the curves $K_{I}$ and $K_{M}$ (Figure 6), hence, the term interacting complexity amplitudes. In Figure 6, the region bounded by segments of the $K_{I}$ and $K_{M}$ spectra and the *x*-axis (individual amplitude axis) is designated as the KC plane. Key variables—including overall complexity, individual system complexities, and spatial patterns within the KC plane—can be directly compared using a normalized two-dimensional framework (0–1 scale). Normalizing the time series places all metrics on a uniform scale, eliminating distortions from differing units or magnitudes. This standardization enables precise evaluation of relationships, patterns, and synergies without scale-related bias [21].

### 3.4. Histogram of Relative Change of Complexity (RCC)

The *histogram of relative change of complexity* refers to a specialized analytical tool that extends traditional histograms to capture multi-scale complexity shifts in systems with interacting components. While the exact terminology varies, its core function is tracking structural. Some of them are: Statistical Physics (analyzing macrostates, gaps, and multi-scale complexity in datasets), Information Theory (balancing coding/decoding costs, entropy-based comparisons), Biology/Bioinformatics (revealing mechanistic/biological information from data distributions), Network Science (comparing network structural complexity, diversity scoring), manufacturing (monitoring process variability, detecting process shift), and Social Sciences (comparing group differences, analyzing temporal changes in complexity). Precisely because of the capabilities of RCC, we decided to use this method in the analysis of streamflow time series, since this type of time series has patterns that are quite difficult to detect using other informational measures when it comes to complexity. Histograms of RCC against normalized interacting complexity amplitudes can reveal the following patterns: (1) Directionality of complexity shifts: Quantifying whether system complexity increases or decreases as interaction strength varies. (2) Distribution characteristics: Distinguishing uniform, skewed, or bimodal distributions of relative changes, which indicate underlying system dynamics (e.g., phase transitions or multi-stability). (3) Amplitude-dynamics relationships: Mapping how the rate or magnitude of interacting complexity amplitudes correlates with emergent complexity patterns (e.g., nonlinear thresholds or hysteresis effects).

One way to reveal the relationship between the overall complexity of a system and the complexities of its individual components is to quantify the interrelationship between the master (overall) complexity of the system and that of its components within an interval of normalized complexity amplitudes, using steps as follows:

(1) Map ($K_{I},K_{M}$) points onto overlapping two-dimensional systems (Figure 5).
(2) Identify the KC plane by removing points outside its boundaries (in this example, represented by black for the master component and red for the individual components).
(3) Calculate the mean complexity ($K_{C}$) values for the master component (A) and individual components (B, C, D) within a defined interval of the KC plane. For the master component $K_{C}^{\left(A\right)}=\frac{1}{N}\sum_{i=1}^{N}K_{C}^{\left(A\right)}(i)$, where N is the number of data points in the interval, while for individual components $K_{C}^{\left(X\right)}=\frac{1}{N}\sum_{i=1}^{N}K_{C}^{\left(X\right)}(i)$, where X = B, C, D.
(4) Compute the relative change of complexity (RCC) between the overall complexity (A, initial value) and the individual complexities (B, C, D, final values)
  $${RCC}_{X}=\frac{K_{C}^{\left(X\right)}-K_{C}^{\left(A\right)}}{K_{C}^{\left(A\right)}}$$
  where X = B, C, D.

This quantifies the percentage increase or decrease in complexity relative to the master component.

**Figure 7:**
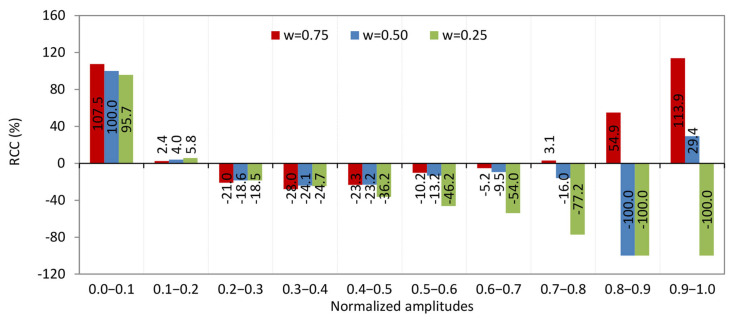
Histogram of relative change of complexity (RCC) against normalized interacting complexity amplitudes in the KC plane.

The histogram of relative change of complexity (RCC) against normalized interacting complexity amplitudes typically shows how the complexity of a system changes relative to different levels of complexity of interacting amplitudes (Figure 7). This kind of histogram visualizes the distribution of relative changes in complexity as a function of varying interaction strengths or amplitudes [22]. Let us illustrate how to interpret the histogram of the considered formal system for the interval 0.8–0.9 (Figure 7).

A positive RCC of A relative to B means that as the complexity of B increases, the complexity of the overall system A increases more than proportionally, or A is more complex relative to B. In other words, B contributes to increasing the overall complexity of A.

A negative RCC of A relative to C and D means that as the complexity of C or D increases, the relative complexity of A decreases compared to them. This could indicate that C and D contribute to simplifying or structuring the system in a way that reduces the overall complexity of A relative to their own complexities. Alternatively, A may share redundancies or dependencies with C and D, which reduces its relative complexity when compared to these components [22].

**Figure 8:**
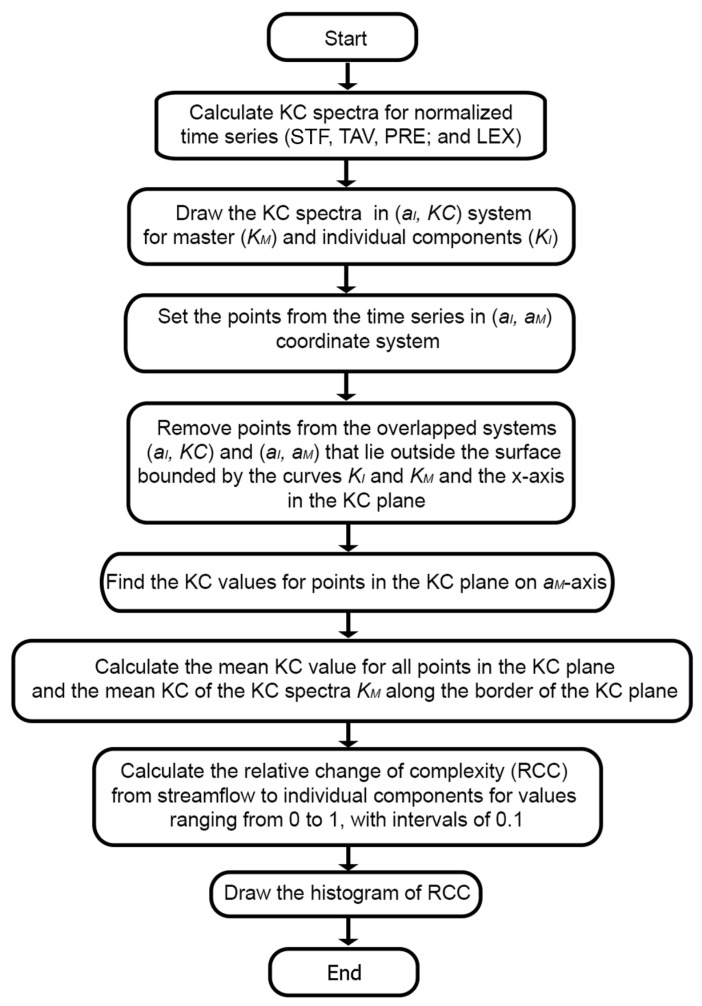
Flow chart of the methodology used in the study.

In essence, this pattern suggests that component B is a complexity-driving factor for system A, increasing its algorithmic information content, while components C and D act in a way that reduces or constrains the overall complexity of A, possibly by introducing regularities or dependencies that lower the effective algorithmic complexity of the system. For clarity, a simple flowchart of the methodology used in this study is given in Figure 8.

## 4. Results and Discussion

Before we move on to the details of the discussion of the obtained results, we will reiterate the focus of this study, elaborated as follows. With the proposed method, we intend to determine how the complexity of mean monthly river flows (STF) on a continental scale (territory of the U.S.) depends on the complexity of the mean monthly values of temperature (TAV), precipitation (PRE), and the parameter of the dynamics (LEX) of those flows over the period 1950–2015. The discussion will be carried out through the following steps: analyzing $\left(K_{I},K_{M}\right)$ points in overlapping two-dimensional planes, localizing $\left(K_{I},K_{M}\right)$ points within the KC plane, examining their distribution in the RCC histogram, and analyzing RCC across intervals of individual amplitudes ranging from 0 to 1 in defined segments. Based on these results, we analyzed and discussed the complexity patterns of U.S. rivers associated with each interval of normalized amplitudes.

### 4.1. Analyzing Location of (K_I_,K_M_) Points in Overlapping Two-Dimensional Planes

**Figure 9:**
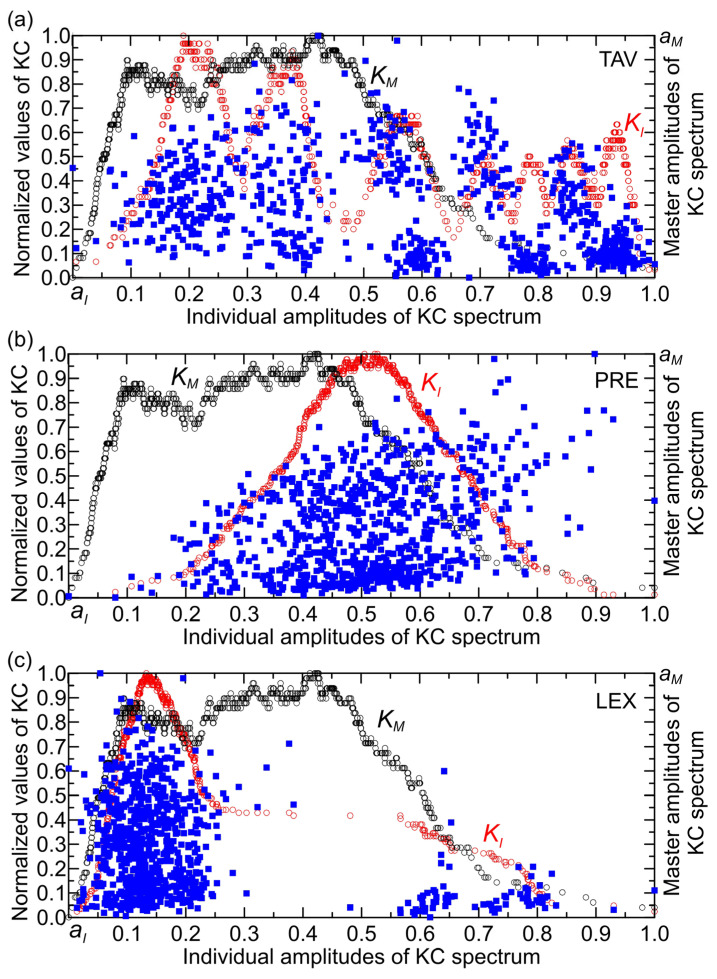
The points $\left(K_{I},K_{M}\right)$ are plotted on overlapping two-dimensional planes for the STF, and (**a**) TAV, (**b**) PRE, and (**c**) LEX time series, with a total N =792 points across all series.

For the TAV time series (with a maximum value of 25.7 °C), the KC spectrum exhibits characteristics of damped oscillations, where their amplitudes decrease progressively from lower to higher normalized individual amplitudes (Figure 9a). The points in the KC plane are predominantly clustered at normalized individual amplitudes below 0.45, indicating a denser concentration in this range. Within the interval of 0.45 to 0.8, the distribution reveals one group of points with relatively high KC values, alongside two other groups characterized by lower KC values. The number of TAV points in the KC plane was 453. From the same figure, it can be seen that the largest number of points lying outside the boundaries of the KC plane (339 points) occurs in the complexity amplitude intervals (0.40, 0.55) and amplitudes higher than 0.65. These correspond to monthly temperature ranges of (10, 14) °C and (17, 26) °C, respectively. Points outside the KC boundaries cluster mainly between 0.40 and 0.55 individual amplitudes, likely because this range corresponds to specific intervals of monthly temperatures (10, 14) °C where the relationship between temperature and streamflow behavior changes, causing deviations from the expected KC plane limits. This clustering suggests that, within this individual amplitude interval, system dynamics or external influences produce more variability or nonlinear effects that cause points to fall outside the KC boundaries. In contrast, at higher individual amplitudes (above 0.7, i.e., corresponding to temperatures higher than 17 °C), the KC values are generally low, indicating less influence of temperature on streamflow, which aligns with the observation that temperature impact diminishes at higher individual amplitudes. Thus, the clustering reflects the interplay between complexity amplitude and temperature effects on the system’s behavior, with the 0.40–0.55 range marking a transition zone of more pronounced deviations.

Higher temperatures in the U.S. generally lead to altered streamflow regimes with shifts in the timing and magnitude of high flows (higher individual amplitudes), often causing reduced streamflow in many regions due to increased evapotranspiration and changes in snowmelt. However, the response varies regionally and seasonally, with some areas experiencing increased high flow events in certain seasons. This complex interplay highlights the critical role of temperature in shaping streamflow dynamics across the U.S. [23,24,25]. However, these studies, along with most others, do not explicitly address the influence of temperature on streamflow complexity, particularly in terms of quantitative assessment. Although empirical evidence suggests that temperature contributes to streamflow complexity, its impact at higher flow amplitudes appears to be less pronounced due to lower KC values and the dominant effects of other hydrological processes. Despite this, the quantification of temperature’s role in streamflow complexity remains insufficiently developed and has yet to reach an acceptable level of precision. The position of points outside the KC plane in Figure 9a appears to align well with this empirical observation. However, it is important to recognize that the temperature component influences streamflow complexity synergistically alongside precipitation and streamflow dynamics. In other words, the effect of temperature is more clearly revealed when examined through the RCC histogram.

Analyzing the PRE time series, which has a maximum value of 117.7 mm, reveals that the shape of the KC spectrum resembles a bell curve (Figure 9b). The points are predominantly concentrated at normalized individual amplitudes within the interval (0.1, 0.8), with KC values reaching up to 0.7. The KC plane contains 623 PRE points, while 169 points lie outside its boundaries. Precipitation influences the KC of streamflow by affecting the variability and randomness of the streamflow time series. Changes in precipitation patterns, such as shifts from snow to rain or variations in precipitation intensity and frequency, alter the input to the hydrological system, which in turn impacts the complexity of the resulting streamflow signals. Higher variability or irregularity in precipitation tends to increase the randomness and thus the KC of streamflow, reflecting a more complex and less predictable system. Conversely, more uniform or stable precipitation can lead to decreased KC, indicating more regular and predictable streamflow behavior [26]. The KC and its derivatives provide quantitative insight into how precipitation variability contributes to the degree of randomness and predictability in streamflow, complementing other measures like the Lyapunov exponent, which assesses predictability horizons [27].

Figure 9c, derived from the LEX time series (maximum value: 0.25), illustrates the KC. The spectrum presents as a right-skewed, asymmetrical curve, peaking at a KC value of 0.15 normalized individual amplitudes. It is seen that there are two clusters of points—one with a very dense concentration in the interval of individual amplitude up to 0.25. The second cluster is characterized by a low concentration of points for individual amplitudes above 0.55. Notably, there is an apparent gap in the amplitude interval (0.25, 0.55), indicating that the complexity amplitudes of the KC spectra for both the Lyapunov exponent and streamflow do not overlap or interact within this range. This separation suggests distinct dynamical regimes or behaviors between clusters, reflecting a lack of shared complexity characteristics in this specific amplitude interval [27]. The KC plane contains 706 LEX points, while 86 points lie outside its boundaries.

### 4.2. Location of ($K_{I},K_{M})$ Points in the KC Plane and Their Distribution in the RCC Histogram

**Figure 10:**
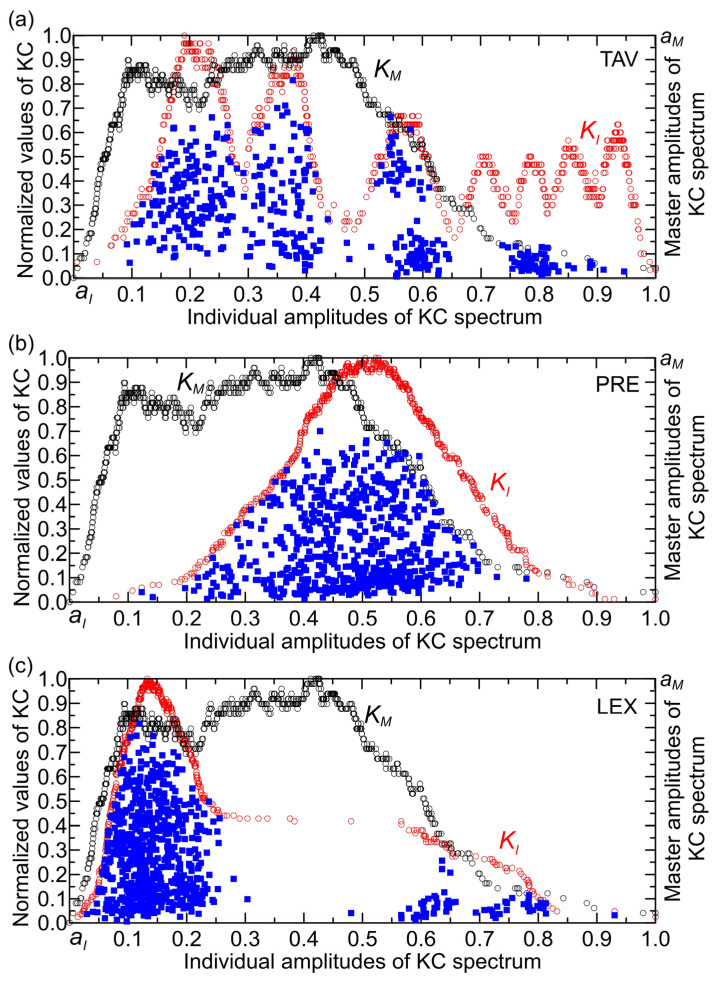
The same as Figure 9, but without points removed from the area outside the KC plane for (**a**) TAV, (**b**) PRE, and (**c**) LEX.

Figure 10 illustrates the locations of points $\left(K_{I},K_{M}\right)$ in the KC plane for the STF, TAV, PRE, and LEX time series. It shows how the amplitudes of overall complexity (STF) and individual complexities (TAV, PRE, and LEX) vary across different intervals: (1) For TAV (Figure 10a), the interval is approximately (0.1, 0.95), with a gap between 0.65 and 0.73, and KC values reaching up to 0.8. (2) For PRE (Figure 10b), the interval spans roughly (0.1, 0.8), featuring a few narrow gaps, with KC values up to 0.7. For LEX (Figure 10a), there are two distinct intervals: one from 0 to 0.3, and another between 0.56 and 0.83. These are separated by a gap in the interval extending from about 0.3 to 0.56 (except the value of 0.47), with a shorter gap between 0.66 and 0.69.

**Figure 11:**
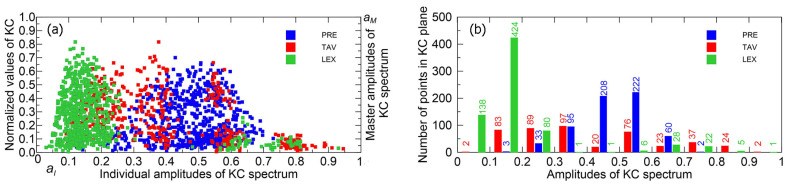
(**a**) Locations of $\left(K_{I},K_{M}\right)$ points in the KC plane for the TAV, PRE, and LEX time series. (**b**) Distribution of the number of points in normalized amplitude intervals for each time series.

In Figure 11a, all points in the KC plane originating from climate drivers (TAV and PRE) as well as from LEX, which is an indicator of system dynamics, are shown. These factors have the greatest influence on the streamflow complexity, both individually and in synergy. The points are grouped into intervals of normalized individual amplitudes, each spanning 0.1 units, ranging from 0 to 1.

Observing Figure 11b, it can be noticed that there are no PRE points in the intervals (0, 0.1), (0.8, 0.9), and (0.9, 1). This pattern can be interpreted as an absence of amplitudes in the KC spectrum of PRE that contribute to its complexity within these intervals.

**Figure 12:**
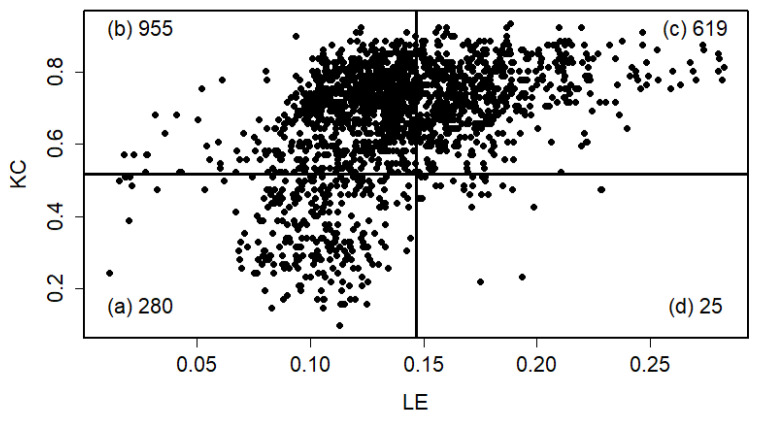
Relationship between Kolmogorov complexity (KC) and Lyapunov exponent (LE) for monthly U.S. streamflow data from 1950 to 2015. Scatter plots display KC versus LE values for gauge stations across four quadrants of the plot area: (**a**) lower-left (LL), (**b**) upper-left (UL), (**c**) upper-right (UR), and (**d**) lower-right (LR). Numbers inside rectangles indicate the total count of gauge stations within each quadrant. Reprinted from [16] with permission. Copyright 2023 Elsevier.

For completeness, the following discussion uses the scatter plot of Kolmogorov complexity (KC) versus Lyapunov exponent (LEX) for U.S. rivers (Figure 12). The plot area is divided into four rectangles by two lines—LEX = 0.146 (parallel to the *y*-axis) and KC = 0.516 (parallel to the *x*-axis)—to enhance visualization for further analysis. These threshold values represent the means of the maximal and minimal KC and LEX values across all gauge stations. The figure reveals that streamflow time series from all gauge stations exhibit a mixture of persistent chaos and high randomness. Randomness reflects unpredictability due to a lack of sufficient information, whereas chaos lies between randomness and predictability. A key characteristic of chaotic streamflow is its inherent predictability. This scatter plot, which displays a distinctive boomerang-shaped pattern, highlights the inherent difficulty of reliably predicting U.S. river streamflow. The analysis further indicates that river discharge behavior varies with the temporal scale: it appears random and non-chaotic at daily and seasonal intervals, yet demonstrates chaotic dynamics at the monthly scale [28]. Figure 12 illustrates that all gauge stations located in the UL quadrant exhibit positive high LEX values, with 83.7% of these stations also displaying KC values above 0.516—placing them in both the UL and UR quadrants. In contrast, the lower quadrants (LL and LR) are predominantly characterized by lower randomness and lower Lyapunov exponent (LE) values. The ratio of gauge stations with low to high randomness is approximately 1:5, underscoring the limited predictability of U.S. river streamflow.

### 4.3. Analysis of RCC Across Intervals of Individual Amplitudes

Before analyzing the patterns across intervals of individual amplitudes, it is important to clarify a point regarding the intervals (0, 1), (0.8, 0.9), and (0.9, 1), particularly concerning the smallest and largest amplitudes. The RCC value for precipitation is −100%, indicating that there are no data points for the KC of precipitation within these intervals on the KC plane. This absence is clearly reflected in the histogram shown in Figure 11b. U.S. rivers do exhibit chaotic behavior and complexity in their streamflow, as quantified by measures such as the Lyapunov exponent and Kolmogorov complexity. However, these complex and chaotic dynamics are inherently linked to precipitation and other environmental factors; they do not occur independently of precipitation.

The absence of points within certain amplitude intervals on the KC plane, as previously emphasized, indicates that these amplitude ranges do not contribute to the complexity of precipitation (PRE) in the KC spectrum. This situation can arise when time series elements are averaged over many data points, resulting in KC spectrum values for streamflow that approach zero—such as the KC values reported in Table 2 for the intervals (0.8, 0.9) and (0.9, 1). In natural settings, this scenario corresponds most closely to regions with low or very low precipitation. Therefore, when analyzing patterns within these intervals, we will take up this assumption.

**Figure 13:**
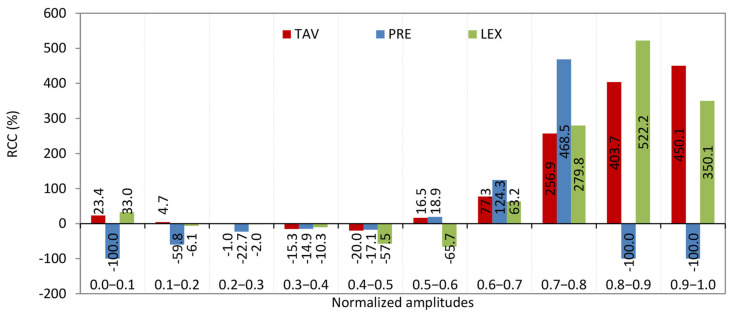
Histogram of relative changes in complexity (RCC) of U.S. river streamflow from 1950 to 2015, compared against temperature (TAV), precipitation (PRE), and Lyapunov exponent (LEX) values, presented at 0.1 intervals.

*0.0–0.1*. From Figure 13, it is seen that the RCC from streamflow to the TAV and LEX is positive, but it is slightly higher for LEX. This means: (1) both temperature and LEX time series are more complex (less regular) than streamflow, since a positive relative change indicates an increase in complexity; and (2) the increase in complexity is slightly greater when comparing streamflow to the Lyapunov exponent than to temperature. The Brazos River in Texas, particularly at the Graford station, exhibits streamflow dynamics where complexity is influenced by temperature and the Lyapunov exponent. Western U.S. Rivers: Rivers in the Southwest, such as the Colorado River tributaries, show similar behavior, where arid climates magnify temperature-streamflow coupling and dam operations elevate LEX [27].

From Figure 13, it is evident that the RCC from streamflow to TAV and LEX is positive, with RCC slightly higher for LEX. This indicates that (1) both TAV and LEX time series are more complex (less regular) than streamflow, as a positive RCC reflects an increase in complexity; and (2) the increase in complexity is marginally greater when comparing streamflow to LEX than to TAV. The Brazos River in Texas, particularly at the Graford station, exemplifies streamflow dynamics where complexity is influenced by both TAV and LEX. Similarly, rivers in the western U.S., such as tributaries of the Colorado River, exhibit comparable behavior, where arid climates intensify the coupling between temperature and streamflow, and dam operations contribute to elevated LEX values [27].

*0.1–0.2*. The described pattern of relationships among streamflow complexity and the complexities of TAV, PRE, and LEX can be interpreted as follows. (1) A small positive RCC of streamflow to the KC of temperature means that increases in temperature complexity slightly increase the streamflow complexity. This suggests that temperature variations have a minor but direct influence on making streamflow more complex or unpredictable. (2) A small negative RCC of streamflow to the KC of the LEX indicates a weak inverse relationship between the chaotic dynamics of the system and streamflow complexity. As the system’s chaotic behavior (LEX complexity) increases, streamflow complexity slightly decreases, implying some stabilizing effect. (3) A negative relative change with a much larger absolute value of streamflow complexity to the KC of precipitation means that PRE complexity has a strong inverse impact on streamflow complexity. In other words, as precipitation patterns become more complex or unpredictable, streamflow complexity significantly decreases. This highlights precipitation as the dominant driver influencing streamflow complexity.

U.S. rivers where precipitation is the dominant driver of streamflow variability and complexity—such as those in regions with highly variable rainfall patterns—are likely to exhibit these traits. For example: (1) Rivers in the southeastern U.S. and Gulf Coast [29,30], where precipitation variability strongly influences streamflow. (2) Some rivers in Texas, including parts of the Brazos River basin, where precipitation dominates streamflow complexity, while temperature and chaotic dynamics have smaller effects. Rivers characterized by hydrology strongly driven by precipitation and moderately influenced by temperature—such as the Brazos River and several rivers in the southeastern United States—are likely to exhibit complexity relationships within the (0.1, 0.2) interval range.

*0.2–0.3*. The pattern observed in this interval reveals nuanced interactions among temperature, precipitation, and chaotic dynamics (LEX) that subtly influence the complexity of streamflow. (1) The negative and very small RCC of streamflow to temperature complexity suggests that variations in temperature randomness have minimal and slightly inverse effects on streamflow complexity. In other words, increasing temperature complexity slightly reduces streamflow complexity, but the effect is negligible. (2) Similarly, the negative and very small RCC of streamflow to LEX complexity implies that changes in the chaotic behavior (LEX) have a minimal and slightly inverse influence on streamflow complexity. (3) The negative RCC of streamflow to PRE complexity with a larger absolute value than the other two indicates that precipitation complexity has a more substantial inverse effect on streamflow complexity. This means that as precipitation randomness increases, streamflow complexity tends to decrease more noticeably. Given the values LEX = 0.2 and KC = 0.96 for streamflow (UR quadrant in Figure 12), this points to a system with very high randomness (complexity near 1) but moderate chaos (LEX = 0.2), suggesting a highly irregular but somewhat predictable flow pattern in the short term. This pattern implies that in these rivers: (i) Streamflow complexity is driven more strongly by precipitation variability than by temperature or chaotic dynamics. (ii) The system is highly complex (KC = 0.96), indicating very irregular flow patterns. (iii) Moderate chaos limits predictability to less than about five months [16].

U.S. rivers with these characteristics are typically found in the arid and semi-arid regions of the U.S., where precipitation is highly variable and episodic, temperature variability has a lesser direct impact on streamflow complexity, and moderate chaotic dynamics emerge from nonlinear hydrological processes characteristic of these climates. Southwestern rivers such as the Colorado River [31] and its tributaries, where precipitation is sporadic and highly variable, leading to high streamflow complexity and moderate chaos. These rivers have been observed to have lower predictability horizons (under 5 months) and high KC values near 0.9 or above [16]. Other rivers in arid basins of Texas and New Mexico also fit this profile, with high KC and LEX around 0.2, reflecting the dominance of precipitation variability in driving streamflow complexity. This aligns with findings that arid southwestern U.S. rivers have the highest complexity and higher LEX compared to other regions, with precipitation playing a dominant role in shaping streamflow dynamics [16,27].

*0.3–0.4.* This pattern of relative changes in KC indicates a −15.3% change in streamflow KC relative to temperature (TAV), a −10.3% change relative to the Lyapunov exponent (LEX) KC, and a −14.9% change relative to precipitation (PRE) KC. These negative values suggest that the complexity of streamflow is somewhat lower than that of temperature (TAV), chaotic dynamics (LEX), and precipitation (PRE). This implies a moderate decoupling, where streamflow dynamics are less complex compared to these environmental drivers. It indicates that streamflow may be influenced by factors that reduce its variability or regularity relative to temperature, chaotic behavior, and precipitation.

Regarding rivers in the U.S. exhibiting these characteristics, studies on U.S. river streamflow complexity reveal that such patterns often occur in regions with relatively stable or subdued hydrological variability. Rivers in areas experiencing moderate drought or low precipitation, where streamflow responses are dampened, tend to show reduced complexity relative to environmental inputs. Examples likely include rivers in semi-arid regions or those affected by human regulation (e.g., dams), where streamflow complexity is constrained. Specific rivers fitting this profile can be found in the following regions: (1) Western and Southwestern Rivers: Colorado River (Lower Basin), Rio Grande (Upper Basin), and Gila River (AZ). (2) Great Plains and Midwest Rivers: Missouri River Basin, Dearborn River (MN), and Platte River (NE). (3) Southern Rivers: Mississippi River, and Texas Rivers (e.g., Guadalupe, San Antonio, Colorado Rivers in Texas). These rivers are located in areas where climatic and anthropogenic factors limit streamflow variability and complexity.

*0.4–0.5*. The pattern in this interval—where the RCC of KC of streamflow to temperature is −20.0%, to LEX is −57.5%, and to precipitation is −17.1%, all negative—indicates a strong inverse relationship between the complexity of streamflow and the complexities of its controlling factors. (1) The negative RCC means that as the complexity of temperature, precipitation, or chaotic dynamics (LEX) increases, the complexity of streamflow decreases. This suggests that increases in environmental or chaotic complexity do not translate into more complex streamflow patterns but rather the opposite. (2) The particularly large negative change with the LEX (−57.5%) implies that chaotic behavior strongly constrains or reduces streamflow complexity, possibly reflecting nonlinear feedbacks or stabilizing mechanisms in the river system. (3) Moderate negative changes with temperature (−20.0%) and precipitation (−17.1%) indicate that climatic variability also inversely affects streamflow complexity but less strongly than chaotic dynamics. (4) This pattern suggests a hydrological system with buffering or regulatory processes—such as watershed characteristics, climatic aridity, or human interventions like dams—that dampen the effect of climatic and chaotic complexity on streamflow.

Regarding U.S. rivers with the aforementioned characteristics, it can be said the following. These rivers, particularly those in the southwestern United States, exhibit a strong inverse relationship between streamflow complexity and the complexities of temperature, precipitation, and chaotic dynamics as measured by the LEX. This pattern reflects the influence of arid climate conditions, mountainous terrain, and significant human interventions such as dam regulation, which collectively buffer or regulate streamflow complexity despite increases in environmental and chaotic variability. Notably, rivers like the Brazos River in Texas exemplify these dynamics, where hydroelectric and other anthropogenic factors contribute to nonlinear feedbacks that reduce streamflow complexity as climatic and chaotic complexities rise [27]. Similar behaviors are observed in other regulated or semi-arid region rivers across the Southwest and parts of the Midwest, Southeast, and Central-East (see Figure 1b), highlighting the interplay between natural and human controls in shaping river flow complexity [32,33].

*0.5–0.6*. The pattern—where the RCC of KC of streamflow to temperature is 16.5%, to LEX is −65.7%, and to precipitation is 18.9%. Temperature and precipitation are climatic drivers, while the LEX measures the predictability or chaotic behavior of the system. (1) A 16.5% relative change in streamflow complexity corresponds to changes in the KC of temperature, indicating a moderate positive sensitivity of streamflow complexity to temperature variability. (2) The strong negative relative change of −65.7% between streamflow complexity and the KC of the Lyapunov exponent suggests that as the chaotic behavior (measured by the LEX) becomes more complex, the complexity of streamflow decreases significantly. This implies that higher unpredictability or chaos in the system reduces the complexity of streamflow patterns. (3) An 18.9% relative change between streamflow complexity and precipitation complexity shows that precipitation variability positively influences streamflow complexity.

A large-scale study analyzed monthly streamflow data from 1879 U.S. rivers (1950–2015) applying KC and LE to assess randomness and chaotic behavior [16]. This study demonstrated that the LEX values for rivers exhibiting the described pattern are positive but generally low, with many rivers having LEX values below 0.149, indicating weak chaotic behavior or moderate predictability. Additionally, KC values are below 0.5, reflecting moderate randomness or complexity in the streamflow time series. Rivers with these characteristics (LL quadrant in Figure 12) are found across various U.S. regions, including the West, Midwest, Southwest, Southeast, and Northeast. Areas with mean monthly streamflow ranging from 14.2 to 70.8 m^3^/s and from 70.8 to 240.7 m^3^/s consistently show KC and LEX values within these intervals, suggesting that these rivers commonly exhibit moderate complexity and weak chaotic dynamics [16].

*0.6–0.7*. There are no specific documented U.S. rivers directly identified with the exact combination of characteristics of this interval—relative changes of streamflow complexity to temperature (77.3%), precipitation (124.3%), and Lyapunov exponent (63.2%), with a LEX of 0.152 and KC of 0.250—in the available research entries. However, based on ongoing work in hydrology and complexity theory, rivers exhibiting moderate and high LEX values ranged from ~0.15 up to 0.25 and KC around in the interval from 0 to 0.5, with strong sensitivity of streamflow complexity to precipitation and temperature, are likely to be found in regions with significant climate variability and anthropogenic influence [16]. These include rivers in the Southwest, Midwest, and other diverse U.S. regions where complex interactions between climate drivers and streamflow dynamics occur.

*0.7–0.8*. This pattern is particularly noteworthy. Using the calculated KC values, a machine learning model was employed to quantify the relationship between percentage changes in precipitation KC and corresponding changes in streamflow KC [34]. The resulting sensitivity coefficient of 468.5% represents the regression slope, indicating that precipitation KC exerts the strongest influence among the variables analyzed. In practical terms, a 1% increase in precipitation KC corresponds to a 468.5% increase in streamflow KC. This influence is followed, in descending order, by the Lyapunov exponent (LEX) and temperature.

U.S. rivers exhibiting these characteristics include: (1) Colorado River (Southwest)—Streamflow KC is primarily driven by precipitation KC due to the region’s extreme aridity and monsoon variability. Additionally, high lagged entropy (LEX) resulting from reservoir operations further influences flow dynamics. (2) Rio Grande (New Mexico/Texas)—Exhibits a remarkable 468.5% sensitivity of streamflow KC to precipitation KC, reflecting its characteristic “boom-bust” hydrology. Snowpack loss and irrigation withdrawals amplify flow randomness, with KC values exceeding 0.55 (LL quadrant in Figure 12). (3) Missouri (River Great Plains)—Displays high LEX-driven chaotic behavior influenced by groundwater interactions and extensive agricultural withdrawals. The 279.8% linkage between LEX and KC highlights the system’s strong sensitivity to initial hydrologic conditions. (4) Tennessee (River Appalachians)—Shows 256.9% sensitivity of streamflow KC to temperature KC, primarily due to warming-induced increases in evapotranspiration. These rivers exemplify “high-complexity hydrology” where climate change and human use amplify flow randomness beyond natural variability.

*0.8–0.9*. U.S. rivers characterized by streamflow strongly influenced by temperature, precipitation, and the Lyapunov exponent—exhibiting a relative change in KC of streamflow to KC of temperature of approximately 403.7%, and to KC of the Lyapunov exponent of about 522.2%, alongside low precipitation—are predominantly located in arid and semi-arid regions where temperature and internal system dynamics primarily drive flow variability. The Colorado River in the Southwest is a prime example. It shows strong sensitivity of streamflow to temperature changes due to snowmelt dynamics and evapotranspiration, with complex flow regulation by reservoirs influencing flow predictability and chaos (high LEX effects) [35]. The Rio Grande (New Mexico/Texas) also fits this profile, where snowpack loss and irrigation withdrawals amplify flow randomness, and streamflow variability is strongly influenced by temperature and system nonlinearities, especially during dry periods with minimal precipitation [36,37]. Other rivers with similar characteristics include some in the Great Plains and arid western basins, where increased winter temperatures and decreased precipitation lead to sporadic streamflow dominated by temperature-driven processes and internal chaotic dynamics [36].

*0.9–1.0.* This pattern has rivers in the U.S. exhibiting high sensitivity to temperature changes, minimal precipitation influence, and chaotic behavior (as implied by LEX and KC metrics) are primarily found in arid and semi-arid regions of the Southwest and Great Plains. The following rivers align with these characteristics: (1) Colorado River Basin. (i) Temperature sensitivity: Studies show runoff declines by 2–9% per °C temperature increase due to reduced snowpack and increased evaporation [31]. (ii) Low precipitation: Arid conditions dominate, with streamflow becoming more sporadic as winter temperatures rise [37]. (iii) Chaotic dynamics: Decadal prediction errors and nonstationarity in streamflow patterns reflect high sensitivity to initial conditions (Lyapunov exponent). (2) Rio Grande Basin: Temperature-driven flow: Similar to the Colorado, temperature increases reduce runoff predictability and amplify complexity. (3) Aridity: Prolonged dry periods and minimal precipitation contribution exacerbate sensitivity. (4) Great Plains Rivers (e.g., Platte, Arkansas): (i) Evaporation dominance: High evaporation rates minimize precipitation impact, making streamflow highly responsive to temperature shifts. (ii) Sporadic flow: Increased days without rainfall intensify chaotic flow patterns [34]. Other rivers do not exhibit the same characteristics as the Colorado River Basin:(i) Humid basins (e.g., the Pacific Northwest) demonstrate lower temperature sensitivity and are more strongly influenced by variations in precipitation. (ii) Snowmelt-dominated systems (e.g., the Columbia River) depend primarily on the timing of precipitation rather than chaotic dynamics to regulate streamflow patterns [38].

## 5. Conclusions

This study investigated how the complexity of individual components—temperature, precipitation, and the Lyapunov exponent—impacts the overall complexity of streamflow. To do this, we used Kolmogorov complexity (KC) metrics, specifically the Kolmogorov complexity spectrum (KC spectrum) and Kolmogorov complexity plane (KC plane). These measures were applied to monthly averaged streamflow time series from 1879 gauge stations on U.S. rivers, covering the period 1950–2015.

For the same period, two monthly averaged time series—temperature and precipitation—were selected to represent individual components of streamflow within this complex physical system. The KC spectrum and KC plane have proven to be effective tools for quantifying the complexities inherent in streamflow as well as its environmental and dynamic components. Using these metrics, we calculated normalized Kolmogorov complexity (KC) spectra for each position within the KC plane, visualizing the master amplitudes interactively alongside individual amplitudes on overlapping two-dimensional planes. Additionally, we quantified the relative change in complexity (RCC) from streamflow to individual components, using values from 0 to 1 at intervals of 0.1.

From the RCC histogram, distinct complexity patterns emerged across U.S. rivers for all intervals examined. These characteristic patterns suggest that streamflow dynamics can be more effectively anticipated using KC-based complexity indicators. Future research should focus on integrating these complexity measures into predictive models and operational tools to enhance the management and forecasting of river flow under varying environmental conditions.

## Figures and Tables

**Table 1:** Descriptive statistics of the time series representing the mean monthly values of streamflow (STF), temperature (TAV), precipitation (PRE), and the Lyapunov exponent (LEX), averaged across 1879 gauge stations on U.S. rivers for the period 1950–2015.

	STF	TAV	PRE	LEX
Mean	147.79	12.33	67.52	0.13
Median	134.77	12.69	67.51	0.12
Min	22.01	−4.53	15.25	0.10
Max	456.66	25.71	117.71	0.25
SD	84.90	8.41	14.57	0.03
Q1	70.48	4.65	58.19	0.12
Q3	211.23	20.42	76.33	0.13
Kurtosis	2.58	1.61	3.29	8.13
Skewness	0.58	−0.07	0.01	2.46

**Table 2:** Calculated Kolmogorov complexity (KC) values for streamflow (KC_STF), temperature (KC_TAV), precipitation (KC_PRE), and Lyapunov exponent (KC_LEX) are presented in the Kolmogorov complexity plane (KC plane), along with relative complexity changes (RCC) of STF compared to TAV (RCC_TAV), PRE (RCC_PRE), and LEX (RCC_LEX) at 0.1 intervals. Empty boxes indicate the absence of amplitudes in the KC spectrum of PRE that contribute to its complexity within those intervals. A value of—100% indicates that there are no data points for the KC of precipitation within these intervals on the KC plane.

Interval	KC_STF	KC_TAV	KC_PRE	KC_LEX	RCC_TAV	RCC_PRE	RCC_LEX
0.0–0.1	0.579	0.714		0.770	23.388	−100%	33.045
0.1–0.2	0.812	0.851	0.327	0.762	4.714	−59.798	−6.132
0.2–0.3	0.836	0.828	0.646	0.819	−0.955	−22.655	−2.025
0.3–0.4	0.910	0.770	0.775	0.816	−15.346	−14.886	−10.307
0.4–0.5	0.912	0.730	0.756	0.388	−19.983	−17.131	−57.475
0.5–0.6	0.645	0.752	0.767	0.221	16.477	18.927	−65.738
0.6–0.7	0.373	0.661	0.836	0.609	77.267	124.288	63.202
0.7–0.8	0.149	0.532	0.847	0.566	256.922	468.524	279.844
0.8–0.9	0.092	0.463		0.571	403.717	−100%	522.241
0.9–1.0	0.041	0.224		0.184	450.123	−100%	350.103

**Figure A1:**
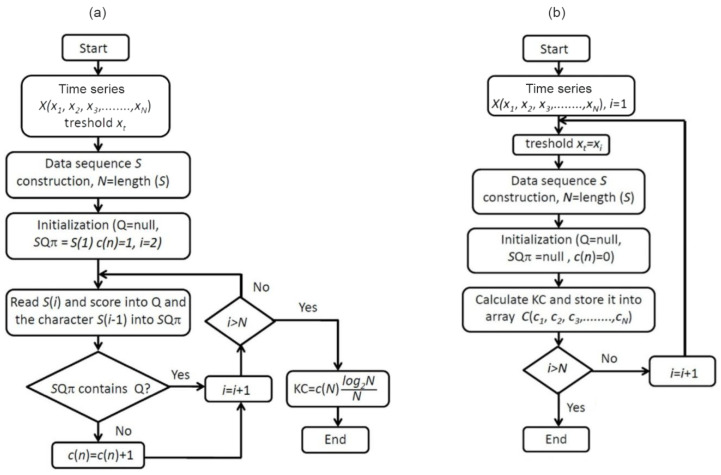
Flowcharts illustrate (**a**) the calculation of Kolmogorov complexity (KC) and (**b**) the computation of the Kolmogorov complexity spectrum (KC spectrum) using the LZA. Reprinted from [39] with permission. Copyright 2025 MDPI.

## Data Availability

The raw data supporting the conclusions of this article will be made available by the authors on request.

## Abbreviations

The following abbreviations are used in this manuscript:

CONUS	Contiguous United States
EPA	U.S. Environmental Protection Agency
HUC8	Hydrologic Unit Code 8
KC	Kolmogorov complexity
LE	Lyapunov exponent in caption of Figure 12
LEX	Time series of Lyapunov exponent
LL	Lower-left
LR	Lower-right
LZA	Lempel–Ziv algorithm
NHD	National Hydrography Dataset
NID	National Inventory of Dams
PRE	Time series of monthly precipitation
RCC	Relative change in complexities
STF	Time series of monthly streamflow
TAV	Time series of monthly temperature
UL	Upper-left
UR	Upper-right
USGS	U.S. Geological Survey

## Appendix A

### Calculating Kolmogorov Complexity (KC) and Kolmogorov Complexity Spectrum (KC Spectrum) Using the Lempel–Ziv Algorithm (LZA)

*Kolmogorov complexity*. Kolmogorov complexity measures the minimal computational resources needed to specify an object, providing a foundational concept in algorithmic information theory. Formally, for a universal Turing machine $U\left(p\right)=x$ the Kolmogorov complexity $K\left(x\right)$ of an object x is defined as: $K\left(x\right)=min\left\{\left|p\right|:U\left(p\right)=x\right\},$ where |p| denotes the length of the shortest program p that, when executed by U, outputs x. This captures the intrinsic complexity of x by quantifying its simplest possible description. Because it is a non-computable measure, it is calculated by applying the LZA (Figure A1a).

*Kolmogorov complexity spectrum*. The Kolmogorov complexity spectrum (KC spectrum) of a time series $\left\{x_{i}\right\}$ is constructed by iteratively applying the Lempel–Ziv (LZA) algorithm with dynamically determined thresholds. For a time series $\left\{x_{i}\right\}$ of length N, the KC spectrum $\left\{c_{i}\right\}$ is generated as follows:

1. *Threshold selection*: Each element $x_{tr,k}$ in the threshold series $\left\{x_{tr,k}\right\}$ is set equal to the corresponding element $x_{k}$ in the original time series, i.e., $x_{tr,k}=x_{k}$ for k=1, 2,…,N.
2. *Binarization*: For each threshold $x_{tr,k}$, the original time series is converted into a binary sequence $\left\{S_{i}^{\left(k\right)}\right\}$ where ${\;S}_{i}^{\left(k\right)}=\left\{\begin{array}{ll} 0 & \mathrm{if}\;x_{i}<x_{tr,k}\\ 1 & \mathrm{if}\;x_{i}\geq x_{tr,k} \end{array}\right.\;\mathrm{for}\;i=1,\;2,\ldots ,N.$
3. *Complexity calculation*: The LZA computes the complexity $c_{k}$ for each binary sequence $\left\{S_{i}^{\left(k\right)}\right\}$. (Figure A1b). The resulting sequence $\left\{c_{k}\right\}$, where k=1, 2,…,N, constitutes the KC spectrum of $\left\{x_{i}\right\}$.

## References

[1] Riesener M., Dölle C., Keuper A., Fruntke M., Schuh G. (2021). Quantification of complexity in cyber-physical systems based on key figures. Procedia CIRP.

[2] Sinha K., Suh E.S., De Weck O.L. (2018). Integrative Complexity: An Alternative Measure for System Modularity. J. Mech. Des..

[3] Kumari U., Upadhyaya S. (2011). An Interface Complexity Measure for Component-based Software Systems. Int. J. Comput. Appl..

[4] Gomes V.M., Paiva J.R.B., Reis M.R.C., Wainer G.A., Calixto W.P. (2019). Mechanism for Measuring System Complexity Applying Sensitivity Analysis. Math. Probl. Eng..

[5] Akundi A., Smith E. (2021). Quantitative Characterization of Complex Systems—An Information Theoretic Approach. Appl. Syst. Innov..

[6] Mediano P.A.M., Rosas F.E., Farah J.C., Shanahan M., Bor D., Barrett A.B. (2022). Integrated information as a common signature of dynamical and information-processing complexity. Chaos.

[7] Golan A., Harte J. (2022). Information theory: A foundation for complexity science. Proc. Natl. Acad. Sci. USA.

[8] Stosic T., Telesca L., de Souza Ferreira D.V., Stosic B. (2016). Investigating anthropically induced effects in streamflow dynamics by using permutation entropy and statistical complexity analysis: A case study. J. Hydrol..

[9] Patil R., Wei Y., Pullar D., Shulmeister J. (2022). Sensitivity of streamflow patterns to river regulation and climate change and its implications for ecological and environmental management. J. Environ. Manag..

[10] Suriano M., Caram L.F., Rosso O.A. (2024). Daily Streamflow of Argentine Rivers Analysis Using Information Theory Quantifiers. Entropy.

[11] Mihailović D.T., Mimić G., Nikolić-Đorić E., Arsenić I. (2015). Novel measures based on the Kolmogorov complexity for use in complex system behavior studies and time series analysis. Open Phys..

[12] Mihailović D., Singh V. (2024). Information in complex physical systems: Kolmogorov complexity plane of interacting amplitudes. Phys. Complex Syst..

[13] Miller M.P., Carlisle D.M., Wolock D.M., Wieczorek M. (2018). A Database of Natural Monthly Streamflow Estimates from 1950 to 2015 for the Conterminous United States. J. Am. Water Resour. Assoc..

[14] Vose R.S., Applequist S., Squires M., Durre I., Menne M.J., Williams C.N., Fenimore C., Gleason K., Arndt D. (2014). Improved Historical Temperature and Precipitation Time Series for U.S. Climate Divisions. J. Appl. Meteorol. Climatol..

[15] Ahrens B. (2006). Distance in spatial interpolation of daily rain gauge data. Hydrol. Earth Syst. Sci..

[16] Mihailović D.T., Malinović-Milićević S., Han J., Singh V.P. (2023). Complexity and chaotic behavior of the U.S. Rivers and estimation of their prediction horizon. J. Hydrol..

[17] Rosenstein M.T., Collins J.J., De Luca C.J. (1993). A practical method for calculating largest Lyapunov exponents from small data sets. Phys. D Nonlinear Phenom..

[18] Shapour M. (2009). LYAPROSEN: MATLAB Function to Calculate Lyapunov Exponent.

[19] Lempel A., Ziv J. (1976). On the complexity of finite sequences. IEEE Trans. Inf. Theory.

[20] Mihailović D., Kapor D., Crvenković S., Mihailović A. (2023). Physics of Complex Systems: Discovery in the Age of Gödel.

[21] Asesh A., Biele C., Kacprzyk J., Kopeć W., Owsiński J.W., Romanowski A., Sikorski M. (2021). Normalization and bias in time series data. Digital Interaction and Machine Intelligence.

[22] Takeda Y., Kawano K., Ma R., Saitoh S., Asao H. (2020). Five patterns of cell signaling pathways associated with cell behavior. bioRxiv.

[23] Constantz J. (1998). Interaction between Stream Temperature, Streamflow, and Groundwater Exchanges in Alpine Streams. Water Resour. Res..

[24] Botero-Acosta A., Ficklin D.L., Ehsani N., Knouft J.H. (2022). Climate Induced Changes in Streamflow and Water Temperature in Basins across the Atlantic Coast of the United States: An Opportunity for Nature-Based Regional Management. J. Hydrol. Reg. Stud..

[25] Wootten A.M., Martin E., Randklev C.R., Smith R. (2023). Projected Changes to Streamflow and Stream Temperature in Central Texas: How Much Will the River Flow?. Earth Interact..

[26] Dey P., Mujumdar P. (2021). On the statistical complexity of streamflow. Hydrol. Sci. J..

[27] Mihailović D.T., Nikolić-Đorić E., Arsenić I., Malinović-Milićević S., Singh V.P., Stošić T., Stošić B. (2019). Analysis of daily streamflow complexity by Kolmogorov measures and Lyapunov exponent. Phys. A Stat. Mech. Appl..

[28] Adab F., Karami H., Mousavi S.F., Farzin S. (2018). Application of Chaos Theory in Modeling and Analysis of River Discharge under Different Time Scales (Case Study: Karun River). Phys. Geog. Res..

[29] Anandhi A., Crandall C., Bentley C. (2018). Hydrologic Characteristics of Streamflow in the Southeast Atlantic and Gulf Coast Hydrologic Region during 1939–2016 and Conceptual Map of Potential Impacts. Hydrology.

[30] Wilbanks K.A., Jackson C.R., Batzer D.P. (2025). Spatial Variability of Long-Term Streamflow Trends in the Southeastern United States. River Res. Appl..

[31] Johnson Z.F., Stuivenvolt-Allen J., Mahan H., Meyer J.D.D., Miksch M. (2024). Upper Colorado River Streamflow Dependencies on Summertime Synoptic Circulations and Hydroclimate Variability. J. Hydrometeorol..

[32] Chalise D.R., Sankarasubramanian A., Ruhi A. (2021). Dams and Climate Interact to Alter River Flow Regimes Across the United States. Earth’s Future.

[33] Ferrazzi M., Vivian R., Botter G. (2019). Sensitivity of regulated streamflow regimes to interannual climate variability. Earth’s Future.

[34] Soler-Toscano F., Zenil H., Delahaye J.-P., Gauvrit N. (2014). Calculating Kolmogorov Complexity from the Output Frequency Distributions of Small Turing Machines. arXiv.

[35] Vano J.A., Das T., Lettenmaier D.P. (2012). Hydrologic sensitivities of Colorado River runoff to changes in precipitation and temperature. J. Hydrometeorol..

[36] U.S. Geological Survey Climate Change is Already Impacting Stream Flows Across the U.S. U.S. Department of the Interior, 15 March 2023. https://www.usgs.gov/news/climate-change-is-already-impacting-stream-flows-across-us.

[37] Gupta A., Carroll R.W.H., McKenna S.A. (2023). Changes in streamflow statistical structure across the United States due to recent climate change. J. Hydrol..

[38] Patterson N.K., Lane B.A., Sandoval-Solis S., Persad G.G., Ortiz-Partida J.P. (2022). Projected effects of temperature and precipitation variability change on streamflow patterns using a functional flows approach. Earth’s Future.

[39] Mihailović D.T., Malinović-Milićević S. (2025). A Novel Approach to Understanding the Complexity of Precipitation. Atmosphere.

